# “Women are again unsafe”: Preventing violence and poor maternal outcomes during current floods in Pakistan

**DOI:** 10.7189/jogh.13.03005

**Published:** 2023-01-20

**Authors:** Sarmad Muhammad Soomar, Abir Arefin, Salman Muhammad Soomar

**Affiliations:** 1 School of Nursing & Midwifery, Aga Khan University, Pakistan; 2 Western University, London, Ontario, Canada; 3 Department of Emergency Medicine, Aga Khan University, Pakistan

**Figure:**
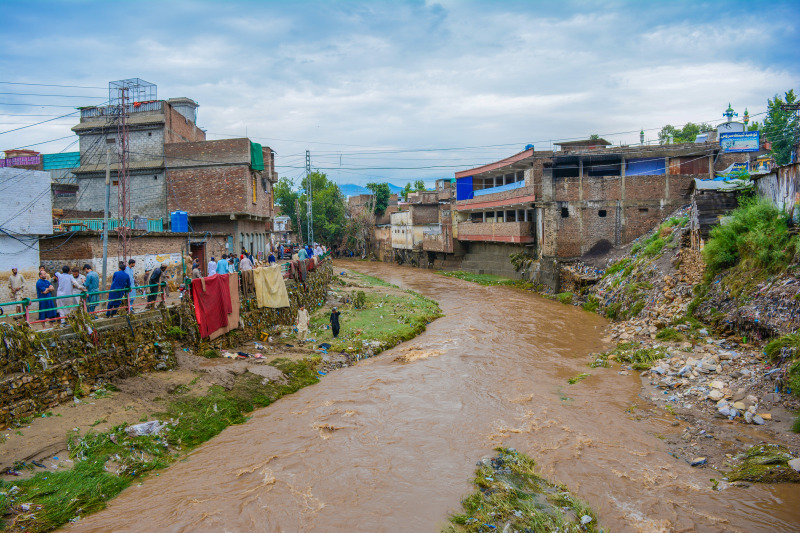
Photo: A scene from current flods in Pakistan. Source: Free to use under PixaBay license (available at: https://pixabay.com/photos/flood-rain-swat-district-pakistan-7411908/?download).

During the ongoing floods in Pakistan, women’s vulnerability to violence and poor maternal outcomes reached an all-time high. According to the United Nations Population Fund (UNFPA), over 6 000 000 women are expecting their pregnancies and around 70 000 of them are expected to go into labor in the upcoming months [1]. Reports routinely warned that health services did not have adequate equipment and teams to perform these deliveries safely. These issues were exacerbated during the floods, as more financial support was needed, as well as a safe environment to relocate pregnant women and support them with quality maternal care. Governmental observations point out that around 1000 essential family health service centers have been negatively affected by the floods, many of them completely ruined. Relocating women in such conditions is a risky intervention, making it a last resort. These isusse, potentiated by crises and floods, require additional attention. Healthcare providers report that if these deliveries are not conducted in a timely and safe manner, women’s lives may be in danger, and their children’s health. Tents, internally displaced camps, or schools are not safe places for pregnant women to deliver their babies, or even to stay at for a period of time. Many of those women are young, either pregnant or displaced with female family members like sisters, mothers, in-laws, or grandmothers. Another vulnerability is access to transportation during floods, which can help them reach camps or even health facilities. Women are reported to be at a higher risk of violence from the people surrounding them due to internal displacement as a result of the floods. A local newspaper reported rape and abuse of women and girls asking truck or rickshaw drivers during the flood crisis for transportation so they could reach their destination for care or safety. The health and safety of women in Pakistan was already a significant issue; this humanitarian crisis has increased their risk 4-fold. Proper research should be carried out to document and report incidences related to maternal health and violence against women during the current floods and the humanitarian crisis. This should also include research on how women’s health and safety should be assured during this time and future crises.

## FLOODS MAKING WOMEN AND GIRLS VULNERABLE

The ongoing floods have devastated Pakistan, leading to a humanitarian crisis. It is important to “leave no one behind in conversations about their health, especially women and girls. Many women of childbearing age are in need of humanitarian assistance. Communication, roads, and bridges in the south of Pakistan have been heavily damaged, which is hindering girls’ and women’s access to healthcare facilities, endangering them and increasing their vulnerability to abuse, violence, and neglect. Factors such as class status, caste, age, sexuality, race, ethnicity, religion, and nationality impact the safety of women in current floods and making them more vulnerable [2].

A humanitarian crisis often leaves women more impoverished, leading to issues such as food insecurity and physical weakness. There are many women expecting to deliver soon and a lack of money impacts their maternal health needs, while food scarcity drastically impacts their nutrition. There is a dire need for food supplies, delivery kits, and funds for transport or necessities in aresa where help has still not arrived. Women in Pakistan and similar countries are already suffering from poor maternal outcomes in general. The crisis has exacerbated this to much higher levels. Lack of access to proper maternal care and essential medications potentially results in pregnancy complications and unsafe birthing conditions.

Displacement affects women and girls’ access to resources such as shelters. Areas like schools or animal yards which are used for their shelter are led by unknown men in charge of their supervision, commuting, and support. Many of them are not safe there, as they are far from their families and male counterparts who are traditionally responsible for them and their safety. Women may not have the resources to rebuild their homes, damaged due to heavy rainfalls and floods, which is why they are forced to stay in these ultimately unsafe spaces. Displaced girls have been observed to be vulnerable; they experience sexual abuse and violence in these shelters and during their commute to their homes or health care facilities. Sexual violence and unprotected sexual encounters are expected to lead to an increase sexually transmitted diseases and injuries [3]. Women who use common contraception are also prone to unwanted pregnancies and more childbirth experiences due to missing their pills.

Women here in floods are also experiencing an increasing budren of the needs of their families and care for their children. Women in displacement camps stop breastfeeding due to a scarcity of energy and poor privacy. Studies have estimated that 75% of displaced persons face such issues due to stress, privacy, and unhygienic conditions [4]. Women have even been reported to stop breastfeeding due to some of these reasons. Various social, emotional, psychological, and physical stressors and community stereotypes in displacement camps force first-time lactating mothers to avoid breastfeeding [5]. Many of these who have moved away from their homes are now relying on formula feed or powdered milk distributed through humanitarian aid.

## PREVENTING VIOLENCE AND POOR MATERNAL HEALTH OUTCOMES

There is a need to prevent the risk of violence and poor maternal outcomes for women affected by the current floods in Pakistan. There is a need for access to skilled birth attendants, newborn care, and supplies to handle the deliveries safely. Special support for preventing gender-based violence and sexual abuse is required. Mental health providers should accompany these women or listen to their stories to lower their stress and help them cope with the situation. Current emergency responses by UNFPA include providing reproductive health service kits called “dignity kits” for women. The psychological impact of violence and manipulation can have a long-term impacts and prevent women from reintegrating into society long after the flood crisis passes. To combat this, gender-based violence prevention strategies and psychosocial support for women and girls must be prioritized. Ensuring women are safe in their shelters and making their commute from homes and to health care facilities and camps safer is also one of their current efforts [6].

Many women and girls who have not yet reached the safety of the displacement camps are often subject to coerced sex, forced marriages, and human trafficking due to their need for shelter, food, and security. Healthcare providers need to reorient their educational and practice frameworks and communication styles to contribute to identifying such social and cultural issues indirectly impacting the women’s health outcomes. There is a need to prevent ignorance in care for rape victims, to include a thorough sexual history, treatment of physical trauma, and to provide emergency contraception and potential care related sexually transmitted infections (STIs). There is a need for better methods for reporting perpetrators of sexual violence and a to identify victims of sexual violence, including young girls who may be at risk [4,7].

**Table 1** provides a summary of the relevant actions for prevention in this regard.

**Table 1:** Summary of actions for prevention

Area	Immediate	Long-term
Preventing poor maternal outcomes	Injection of oxytocin after childbirth to reduce the risk of bleeding: practicing good hygiene and treating early signs of infection; administration of drugs such as magnesium before the onset of convulsions (eclampsia)	Establish a census/registry for the identification of pregnant and postpartum women; tool for identifying patient’s last period/date of conception; tool for identifying high-risk pregnancies; prenatal care centers; post-natal care centers that can perform cesarian sections; administration of tetanus toxoid to pregnant females; availability of clean water for pregnant females
Preventing STIs and unwanted pregnancy	Determine the availability of contraception; document the type of contraception, quantity, and expiration dates; distribution of condoms; emergency contraception; injectable contraception; distribution of medications needed to treat STIs; identify medical units needed to care for HIV-positive women	Access to contraception; safe abortion services; post-abortion care; access to ob-gyn health care services with trained staff in shelters or refugee camps; education sessions in sexual and reproductive health rights; educational supplies on sexual and reproductive health
Preventing violence	Hazard mapping and vulnerability analysis incorporating gender considerations; gender-sensitive emergency relief efforts	Community-based disaster-preparedness projects; disaster training and education programs covering the specific needs of women and men; rapid assessments of health status including gender analysis, reproductive and mental health needs; more information on communicable diseases and nutrition; tool identifying special-risk populations for disaster relief and recovery services; gender training of emergency managers and health service professionals

HIV – human immunodeficiency virus, STI – sexually-transmitted infection

## POLICY RECOMMENDATIONS

Floods have previously affected Pakistan to a varying extent. Some have caused destruction at a smaller scale and impacted a specific part of a province. Many have had a significant impacted at a macroscopical level, affecting all facilities and the functioning of more than one province. One such flood occurred in 2010 and the other one is ongoing in 2022. Women have been unsafe and vulnerable during many of these past floods; they are also vulnerable now, and many have already been impacted and bear the consequences. During such a time of crisis, a disaster relief team equipped to deal with maternal health outcomes and understanding of the sensitivity of women and girls’ reproductive and mental health should be prepared at the country level. This team should work to prevent such situations in advance and plan the strategies for prevention and safety. It should also include health care providers as well, who can support the operationalization regarding these issues to decrease the escalation of such experiences among women and girls if a humanitarian crisis occurs anytime in the future.

One part of a comprehensive disaster care plan should include the availability of contraception for women, determine the type and quantity of contraceptives, determine the availability of condoms to both men and women, promote the use of injectable contraception, establish ob-gyn health care services, and determine the number of educational supplies. Education and resources on STIs should also be included in policy plans, including furthering the education and promotion of condom use and delivering medications to treat STIs. Available medical units available that are adequately equipped to care for HIV-positive women post-disaster should also be identified. The health care providers should also include nurses and midwives who can support maternal outcomes and assist in the healthy and respective birthing process. Curricula on health care and social institutions in Pakistan should incorporate “care and support during humanitarian crises” as an essential study module nationwide.

Research is needed at the local and national levels, including research on violence against women who are disadvantaged and impacted during a crisis. These groups should be identified, perhaps through involving community organizations in this process. Periodical research in Pakistan can also guide the development of policies and practice guidelines to command and operationalize women and girls’ health and safety in humanitarian crises such as floods [8]. These efforts will then never let “women be unsafe again”.
